# Examining Japanese laypeople’s nationality preferences for physicians: a nationwide study

**DOI:** 10.1038/s41598-025-29740-6

**Published:** 2025-11-27

**Authors:** Hirohisa Fujikawa, Hirotake Mori, Yuji Nishizaki, Yuichiro Yano, Toshio Naito

**Affiliations:** 1 Department of General Medicine, Juntendo University Faculty of Medicine, 2-1-1 Hongo, Bunkyo-Ku, Tokyo, 113-8421 Japan; 2 Department of Medical Education Studies, International Research Center for Medical Education, Graduate School of Medicine, The University of Tokyo, Bunkyo-Ku, Tokyo, Japan; 3 Center for General Medicine Education, School of Medicine, Keio University, Shinjuku-Ku, Tokyo, Japan; 4 Division of Medical Education, Juntendo University School of Medicine, Bunkyo-Ku, Tokyo, Japan

**Keywords:** Laypeople, Nationality, Preference, Bias, Public education, Health care, Medical research

## Abstract

This study aimed to investigate physician nationality preferences of Japanese laypeople and the reasons behind the preferences. We performed a nationwide study in May 2025. The participants were monitors of an internet survey company who responded to closed questions regarding nationality preferences across different physician qualities and clinical scenarios, along with open-ended questions regarding their expectations and concerns about care by foreign national physicians. We analyzed responses to the closed questions by descriptive statistics. We compared responses between individuals with prior experience of seeing a foreign national physician and those without such experiences by chi-square test. For free-text responses, we performed inductive content analysis. Among 2004 respondents, approximately half to two-thirds preferred Japanese physicians. Participants with prior experience of seeing a foreign national physician were significantly less likely to indicate a nationality-concordant preference. In free-text responses, 337 participants provided expectations (360 codes) and 938 provided concerns (982 codes). Among concerns, 723 codes were labelled as a language barrier. Participants expressed expectations of the possible strengths of foreign national physicians. This study clarified the nationality preferences of Japanese laypeople in physician selection. The findings have significant implications, including the necessity of reconsidering medical education strategies, public education, and inclusive policy frameworks.

**Supplementary Information:**

The online version contains supplementary material available at 10.1038/s41598-025-29740-6.

## Introduction

Patient-physician interaction serves as the cornerstone of healthcare delivery, and is characterized by mutual trust, respect, and collaboration^1^. Its essential role cannot be overstated in medical education^2,3^. Indeed, this relationship extends back at least as for as the Hippocratic Oath, which established a sacred relationship between patient and physician^4^. Research has indicated a positive association between patient-physician interaction and improved medical outcomes, as well as enhanced patient experience^5–9^. Thus, the patient-physician interaction in large part influences the quality of patient care, and is the foundation of the practice of medicine^10,11^.

The nature of the interaction is influenced by a number of factors, including patient, physician, and social factors^12^. An important line of inquiry concerns concordance between patients and physicians along social identity dimensions. In Western literature, racial/ethnic concordance has been examined extensively^13–15^. Concordance has the potential to enhance communication quality, better trust, and greater patient experience^16,17^, and a considerable body of evidence in the literature in the Western context has shown that patients frequently exhibit a preference for physicians of the same race^18,19^. Furthermore, race-concordant visits are longer and characterized by greater patient positive affect^20^. In contrast, racial disconcordance has been associated with a poorer quality of patient-provider communication^20–26^. These various findings indicate the depth of study of patient racial preferences for physicians in Western countries.

Conversely, however, little is known about whether similar findings would be observed in the Eastern context, particularly in Japan. While population diversity increases in the era of globalization, Japan presents a unique context in this discourse; in official statistics and public discourse, residents are most commonly classified by nationality, and the population is often described as relatively homogeneous on that basis^27^. Additionally, the physician workforce likewise shows limited representation of foreign national doctors^28,29^. However, the number of international medical graduates (IMGs) in Japan has steadily increased, and recent data indicate that approximately 100–200 IMGs pass the Japanese Medical License Examination each year, constituting approximately 1–2% of the total^30^. Despite these shifts, public preferences regarding physicians’ nationality remain underexamined. In contrast to Western countries, Japan is commonly portrayed as a homogeneous society which is known for group harmony and collectivism^31,32^. Considered against the Western literature, this perception indicates a strong need for empirical studies that examine laypeople’s nationality preferences for physicians and their rationales in Japan.

Here, we aimed to elucidate Japanese laypeople’s nationality preferences in physician selection across common clinical scenarios and the reasons behind them. In Western literature, patient-physician racial concordance has been a major focus of inquiry. In Japan, however, nationality rather than race serves as the most salient boundary for social categorization and policy, with official statistics and public discourse usually distinguishing between “Japanese” and “foreign nationals.” Accordingly, the focal point of our study was nationality concordance, a distinction particularly relevant to the Japanese sociocultural setting. We anticipated that the findings of this study would offer insights for medical educators and policy makers, derived from a comprehensive understanding of laypeople’s perspectives in the current era, which is characterized by an increase in diversity within the workforce of the Japanese medical field.

## Methods

### Design, setting, and participants

The study was conducted under a nationwide cross-sectional design in May 2025. It used an anonymous web-based questionnaire targeted laypeople (i.e., non-healthcare staff) living in Japan who were 18 years of age or older. We followed the Checklist for Reporting Results of Internet E-Surveys guidelines^33^. To recruit laypeople, we asked an online survey company (referred to as X company) to distribute the questionnaire. We used quota sampling through the online survey panel. Quotas for age group and residential area were set to approximate the national population distribution. While quotas can improve demographic balance, the sample remains a non-probability, convenience sample because respondents were self-selected from a voluntary sample. Given that our study examined nationality preferences and that the survey panel consists almost entirely of Japanese nationals, we only included individuals with Japanese nationality and excluded those of other nationalities. We also excluded healthcare staff in order to examine the perspectives of laypeople.

Participants registered as monitors of X company were invited to participate in the study via the X company platform. At the beginning of the online questionnaire, we provided a brief explanation of the study. Participants who willingly consented to participating in the study checked the consent box in the questionnaire and completed the questionnaire. Non-respondents received reminder messages from X company several times during the survey period. Although a sample size calculation was not conducted due to the descriptive nature of the study, we set a target of approximately 2000, with reference to previous studies that examined patient preferences for healthcare^34–36^. Accordingly, the survey was closed when the number of responses reached 2000, resulting in a total survey period of 4 days.

### Survey

The questionnaire used in this study was developed by the authors based on previous studies^37–40^ (Supplementary file 1). It consisted of 26 questions which were shown in 17 online pages. Prior to distribution of the questionnaire, it was pilot-tested with a convenience sample of several patients, and the validity was examined using face validity.

Before answering the questionnaire, participants were informed that the survey asked them to consider medical care in Japan, and to compare situations in which the treating physician was Japanese versus of foreign nationality, without assuming that care was provided in an overseas healthcare setting. In the first section, we asked for sociodemographic information. The items asked the participants about their gender, age, living area, marital status, level of education, occupation, annual household income, regularity of visits to medical institutions, and prior experience with care by foreign national physicians.

The second section included a variety of nationality preference items. Four items assessed participants’ preferences regarding physician qualities (i.e., empathy, specialty expertise, communication skills, and being a good listener to patients), and 7 items evaluated participants’ preferences for patient-physician interaction in possible medical scenarios (e.g., taking the patient’s medical history). These 11 items were rated using the following three options: prefer a Japanese physician, prefer a non-Japanese physician, and no preference. In our dataset, values of Cronbach’s alpha were 0.92 for the 4 items regarding physician quartiles and 0.96 for the 7 items regarding possible medical scenarios, both of which indicated good internal consistency reliability^41^.

In the third section, we asked the respondents two optional open-ended questions, the first about their expectations regarding patient care by foreign national physicians and the second about their concerns about patient care by foreign national physicians.

### Data analysis

We analyzed the data of the closed-ended questions by descriptive statistics. Unajusted associations between sociodemographic factors and prior experiences of care by foreign physicians were analyzed by the χ^2^ test for trend. Considering the potential relationship between prior experiences of care by foreign physicians and perceptions of care by foreign national physicians, we also performed subgroup analysis. This analysis compared the responses of individuals with prior experience of being examined by a foreign national physician to those without such experience. The analysis was also conducted using the χ^2^ test. All quantitative analyses were performed using SPSS version 29.0.2.0 (IBM Corp) and *p*-values below 0.05 were considered statistically meaningful.

We analyzed the responses to the open-ended questions by inductive content analysis using Microsoft Excel version 16.96. The definition of content analysis by Haggarty was adopted, namely “a research method which allows the qualitative data collected in research to be analyzed systematically and reliably so that generalizations can be made from them in relation to the categories of interest to the researcher”^42^. Specifically, we conducted the inductive content analysis with reference to previous literature^43–46^: first, the first author undertook an iterative review of the responses, with the objective of fostering a deeper understanding of the data. Second, the initial coding was developed inductively by the first author. Third, all authors participating in reviewing, refining, and discussing the coding scheme. Discrepancies were resolved through repeated discussions until consensus was reached. Fourth, codes were classified into themes and subthemes based on similarities and differences. Fifth, to show its relative importance in the overall picture, the frequency and percentage of each theme and subtheme were calculated using the total number of codes as the denominator, rather than the number of participants, because participants could contribute more than one coded segment. Illustrative quotes were also shown.

### Ethical considerations

We included only individuals that provided their consent to participating in the study by checking the consent box. Following completion of the online questionnaire, participants received points that could be exchanged for cash (a few dozen yen). Ethical approval for this study was obtained from the Ethics Committee of Juntendo University Faculty of Medicine (No. E25-0042). All methods were performed in accordance with the relevant guidelines and regulations.

## Results

### Participants

A total of 2004 laypeople completed the closed-ended items of the questionnaire, and all were included in the final analysis. Of these respondents, 337 provided an open-text response to the expectation question, and 938 provided an open-text response to the concern question. Table 1 demonstrates the participants’ characteristics. A total of 1199 (59.8%) were women, and 1895 (94.6%) had no experience of consulting a foreign national physician. As shown in Table 1, participants with prior experience of being examined by foreign-national physicians were more likely to live in certain regions, have higher educational attainment, visit medical institutions more frequently, and usually attend university hospitals than those without such experience.

**Table 1:** Participants’ profile.

Characteristic	Total (N = 2004)	Have experience of being examined by foreign national physicians (N = 109)	Have no experience of being examined by foreign national physicians (N = 1895)	*P*-value^a^
Gender, N (%) Woman Man Others	802 (40.0) 1199 (59.8) 3 (0.1)	49 (45.0) 60 (55.0) 0	753 (39.7) 1139 (60.1) 3 (0.2)	0.518
Age group, N (%) 18–24 y 25–34 y 35–44 y 45–54 y 55–64 y 65–74 y ≥ 75 y	156 (7.8) 238 (11.9) 298 (14.9) 346 (17.3) 288 (14.4) 328 (16.4) 350 (17.5)	9 (8.3) 15 (13.8) 16 (14.7) 16 (14.7) 12 (11.0) 25 (22.9) 16 (14.7)	147 (7.8) 223 (11.8) 282 (14.9) 330 (17.4) 276 (14.6) 303 (16.0) 334 (17.6)	0.508
Residential area, N (%) Hokkaido and Tohoku Kanto Chubu Kinki Chugoku and Shikoku Kyushu	240 (12.0) 738 (36.8) 230 (11.5) 375 (18.7) 184 (9.2) 237 (11.8)	21 (19.3) 49 (45.0) 8 (7.3) 15 (13.8) 3 (2.8) 13 (11.9)	219 (11.6) 689 (36.4) 222 (11.7) 360 (19.0) 181 (9.6) 224 (11.8)	0.008
Marital status, N (%) Never married Married Divorced or widowed	654 (32.6) 1183 (59.0) 167 (8.3)	26 (23.9) 74 (67.9) 9 (8.3)	628 (33.1) 1109 (58.5) 158 (8.3)	0.119
Level of education, N (%) Less than high school High school Junior college More than or equal to college	50 (2.5) 619 (30.9) 353 (17.6) 982 (49.0)	2 (1.8) 24 (22.0) 13 (11.9) 70 (64.2)	48 (2.5) 595 (31.4) 340 (17.9) 912 (48.1)	0.013
Occupation, N (%) Manager or executive Company employee Self-employed Public employee Employee of other organizations Part-time worker Housewife/househusband Student Unemployed	46 (2.3) 608 (30.3) 159 (7.9) 57 (2.8) 27 (1.3) 257 (12.8) 209 (10.4) 72 (3.6) 569 (28.4)	3 (2.8) 35 (32.1) 11 (10.1) 4 (3.7) 1 (0.9) 10 (9.2) 16 (14.7) 4 (3.7) 25 (22.9)	43 (2.3) 573 (30.2) 148 (7.8) 53 (2.8) 26 (1.4) 247 (13.0) 193 (10.2) 68 (3.6) 544 (28.7)	0.676
Annual household income (million JPY), N (%) < 3.00 (≈ 20,000 US dollar) 3.00–4.99 5.00–6.99 7.00–9.99 ≥ 10.00	608 (30.3) 555 (27.7) 356 (17.8) 287 (14.3) 198 (9.9)	23 (21.1) 30 (27.5) 25 (22.9) 14 (12.8) 17 (15.6)	585 (30.9) 525 (27.7) 331 (17.5) 273 (14.4) 181 (9.6)	0.062
Number of medical visits in the last 6 months, N (%) 0 1 2 3 4 5–9 ≥ 10	584 (29.1) 276 (13.8) 213 (10.6) 204 (10.2) 96 (4.8) 420 (21.0) 211 (10.5)	11 (10.1) 24 (22.0) 11 (10.1) 14 (12.8) 7 (6.4) 23 (21.1) 19 (17.4)	573 (30.2) 252 (13.3) 202 (10.7) 190 (10.0) 89 (4.7) 397 (20.0) 192 (10.1)	< 0.001
Type of medical institution being usually attended, N (%) Clinic Community hospital University hospital	1463 (73.0) 465 (23.2) 76 (3.8)	75 (68.6) 23 (21.1) 11 (10.1)	1388 (73.2) 442 (23.3) 65 (3.4)	0.002

^a^The analysis compared the responses of individuals with prior experience of being examined by a foreign national physician to those without such experience using the χ^2^ test.

### Nationality preferences regarding physicians’ personal qualities and various healthcare scenarios

Table 2 shows the lay participants’ nationality preferences for the specific qualities of physicians and in a variety of medical scenarios. Regarding personal qualities, approximately half to two-thirds of participants showed a preference for Japanese physicians. About half of the participants had no nationality preference in terms of specialty expertise. Participants who had previously consulted a foreign national physician tended to express significantly fewer nationality preferences in physician qualities than those who had not (*p* < 0.01).

**Table 2:** Laypeople nationality preferences for specific qualities of physicians and in various medical scenarios (N = 2004**).**

Physician qualities	Total, N (%)			Have experience of being examined by foreign national physicians, N (%)			Have no experience of being examined by foreign national physicians, N (%)			*P*-value^a^
	Prefer Japanese physician	Prefer foreign national physician	No preference	Prefer Japanese physician	Prefer foreign national physician	No preference	Prefer Japanese physician	Prefer foreign national physician	No preference	
Empathy	1261 (62.9)	14 (0.7)	729 (36.4)	53 (48.6)	6 (5.5)	50 (45.9)	1208 (63.7)	8 (0.4)	679 (35.8)	< 0.01
Specialty expertise	1017 (50.7)	29 (1.4)	958 (47.8)	41 (37.6)	13 (11.9)	55 (50.5)	976 (51.5)	16 (0.8)	903 (47.7)	< 0.01
Communication skill	1313 (65.5)	17 (0.8)	674 (33.6)	59 (54.1)	5 (4.6)	45 (41.3)	1254 (66.2)	12 (0.6)	629 (33.2)	< 0.01
Good listener to the patient	1260 (62.9)	33 (1.6)	711 (35.5)	55 (50.5)	11 (10.1)	43 (39.4)	1205 (63.6)	22 (1.2)	668 (35.3)	< 0.01

Medical care scenario	Total, N (%)			Have experience of being examined by foreign physicians, N (%)			Have no experience of being examined by foreign physicians, N (%)			*P*-value^a^
	Prefer Japanese physician	Prefer foreign national physician	No preference	Prefer Japanese physician	Prefer foreign national physician	No preference	Prefer Japanese physician	Prefer foreign national physician	No preference	
Taking the medical history	1182 (59.0)	15 (0.7)	807 (40.3)	54 (49.5)	2 (1.8)	53 (48.6)	1128 (59.5)	13 (0.7)	754 (39.8)	0.06
Discussing family or psychological problems (e.g., abuse, depression)	1339 (66.8)	21 (1.0)	644 (32.1)	62 (56.9)	6 (5.5)	41 (37.6)	1277 (67.4)	15 (0.8)	603 (31.8)	< 0.01
General physical examination	1075 (53.6)	24 (1.2)	905 (45.2)	40 (36.7)	13 (11.9)	56 (51.4)	1035 (54.6)	11 (0.6)	849 (44.8)	< 0.01
Examination with private body part exposure (e.g., clinical breast examination)	1145 (57.1)	25 (1.2)	834 (41.6)	46 (42.2)	12 (11.0)	51 (46.8)	1099 (58.0)	13 (0.7)	783 (41.3)	< 0.01
General ailments (e.g., cold, stomach pain)	1070 (53.4)	22 (1.1)	912 (45.5)	46 (42.2)	7 (6.4)	56 (51.4)	1024 (54.0)	15 (0.8)	856 (45.2)	< 0.01
Surgical operation	1157 (57.7)	31 (1.5)	816 (40.7)	49 (45.0)	10 (9.2)	50 (45.9)	1108 (58.5)	21 (1.1)	766 (40.4)	< 0.01
Life-threatening conditions	1213 (60.5)	29 (1.4)	762 (38.0)	53 (48.6)	11 (10.1)	45 (41.3)	1160 (61.2)	18 (0.9)	717 (37.8)	< 0.01

^a^The analysis compared the responses of individuals with prior experience of being examined by a foreign national physician to those without such experience using the χ^2^ test.

Among possible medical scenarios also, approximately half to two-thirds of participants exhibited a preference for Japanese physicians. Approximately half of the participants had no nationality preference with regard to general physical examination and general ailments. Further, there was no significant difference in nationality preference regarding the taking of a medical history between those who had previously seen a foreign national physician and those who had not (*p* = 0.06). In addition, we noted that participants who had experience of seeing a foreign national physician tended to express significantly fewer nationality preferences in the medical scenarios, other than taking a medical history, than those who did not have such experience (*p* < 0.01).

### Expectations and concerns towards patient care by foreign national physicians

Tables 3 and 4 present the results of content analysis of responses to the questions regarding expectations (Table 3) and concerns (Table 4) for patient care by foreign national physicians.

**Table 3:** The results of the content analysis of open-ended responses on expectations regarding patient care by foreign national physicians.

Theme, N (%)	Sub-theme, N (%)	Exemplar quotes
Medical skills and knowledge, 222 (61.7)	Skills, 90 (25.0)	“Skills that are not available among Japanese (physicians)”
	Specialized care, 61 (16.9)	“Specialized treatment for cases that are rare in Japan.”
	Knowledge, 51 (14.2)	“Specialized knowledge…in fields that are lagging behind in Japan.”
	New treatment methods, 15 (4.2)	“Introduction of new treatments.”
	Mental care, 4 (1.1)	“Mental care with attention to detail.”
	Research, 1 (0.3)	“Research for the advancement of medicine.”
Communication skills and attitudes, 81 (22.5)	Communication skills, 35 (9.7)	“Humorous and positive.”
	Attitude of being close to patients, 16 (4.4)	“Seems like they would listen to patients attentively.”
	Frank communication, 8 (2.2)	“Foreign physicians seem like they would communicate frankly.”
	Compassion, 7 (1.9)	“Compassionate care for patients”
	Sincerity, 4 (1.1)	“They would take the time to think sincerely.”
	Careful work, 3 (0.8)	“They would respond politely.”
	Language proficiency, 2 (0.6)	“Language proficiency”
	Cheerful attitude, 2 (0.6)	“They would interact in a cheerful manner.”
	Easy to talk to, 2 (0.6)	“A casual, approachable attitude that makes it easy to consult with them.”
	Understanding patients, 1 (0.3)	“Understanding patients.”
	Healing, 1 (0.3)	“Healing.”
Strengths of different cultures, 46 (12.8)	Broad perspective, 21 (5.8)	“Sharing new perspectives and sensibilities stemming from cultural differences”
	Flexible thinking, 8 (2.2)	“Innovative ideas and responses not bound by Japanese stereotypes.”
	Knowledge of overseas medical affairs, 8 (2.2)	“Familiarity with overseas literature and cases.”
	Respect for diversity, 5 (1.4)	“They would help patients from foreign counries in Japan.”
	Reasonable judgement, 3 (0.8)	“With their expertise gained from living overseas, they can provide reasonable care.”
	High motivation, 1 (0.3)	“High motivation.”
Sense of security and trustworthiness, 11 (3.1)	Sense of security, 7 (1.9)	“Sense of security.”
	Trustworthiness, 4 (1.1)	“Trustworthiness.”

Percentages are calculated using the total number of codes generated for expectations (N = 360), not the number of respondents. Multiple codes could be assigned per respondent.

**Table 4:** The results of the content analysis of open-ended responses on concerns regarding patient care by foreign national physicians.

Theme, N (%)	Sub-theme, N (%)	Exemplar quotes
Language barriers, 723 (73.6)	Communication failure, 495 (50.4)	“I am concerned about whether they can understand Japanese sufficiently.”
	Inadequate Japanese language ability, 228 (23.2)	“Miscommunication arising from (inadequate) language ability.”
Cultural incompatibility, 140 (14.3)	Differences in sense of values, 38 (3.9)	“Difficulty in communicating patients’ needs due to differences in sense of values.”
	Lack of understanding of Japanese characteristics, 34 (3.5)	“How much can they understand the unique aspects of Japan and Japanese people?”
	Differences in customs, 28 (2.9)	“I am concerned about whether they can adapt to Japanese customs.”
	Cultural differences, 24 (2.4)	“I am concerned that cultural differences might hinder treatment.”
	Concerns about whether they have the meticulousness unique to Japanese people, 12 (1.2)	“I am concerned about whether they lack meticulous consideration that is valued in Japanese culture.”
	Lack of understanding of the Japanese healthcare system, 4 (0.4)	“(Lack of) understanding of Japanese healthcare system…”
Trustworthiness and safety, 108 (11.0)	Concerns about medical treatment abilities, 38 (3.9)	“I am concernd about their medical technique.”
	Overall concerns, 13 (1.3)	“Vague concerns.”
	Concerns about attitude, 12 (1.2)	“(I am concerned about) too business-like approach during examinations.”
	Concerns about care that takes mental health into consideration, 11 (1.1)	“(I am concerned about their) mental care for patients and their families.”
	Concerns about trustworthiness, 11 (1.1)	“I am concerned about their trustworthiness.”
	Differences in treatment methods, 10 (1.0)	“(I am concerned about) their belief that overseas methods are always correct.”
	Concerns about racial discrimination, 5 (0.5)	“I am concerned about racial discrimination.”
	Concerns about safety and security, 4 (0.4)	“Safety concerns.”
	Concerns about professionalism, 3 (0.3)	“(I am concerned about) violations of regulations.”
	Concerns about privacy, 1 (0.1)	“(I am concerned about) privacy.”
Others, 11 (1.1)	Others, 11 (1.1)	N/A

Percentages are calculated using the total number of codes generated for concerns (N = 982), not the number of respondents. Multiple codes could be assigned per respondent.

As described earlier, a total of 337 participants provided responses to the question regarding the expectations. From these, we generated 360 codes (Table 3). The percentages reported in Table 3 are based on this total number of codes (360). These codes were organized into four themes: “medical skills and knowledge” (e.g., skills), “communication skills and attitudes” (e.g., communication skills), “strengths of different cultures,” (e.g., broad perspective) and “sense of security and trustworthiness” (e.g., sense of security). Of the total 360 codes, 222 (61.7%) were categorized under “medical skills and knowledge.” A considerable proportion of participants expressed an expectation regarding the strengths of foreign national physicians, including their communication skills (35, 9.7%) and broad perspective (21, 5.8%). Exemplar quotes are also shown in Table 3.

As described earlier, a total of 938 participants responded to the question regarding concerns, from which we generated 982 codes (Table 4). The percentages reported in Table 4 are based on this total number of codes (982). These codes were organized into four themes: “language barriers” (e.g., communication failure), “cultural incompatibility” (e.g., differences in sense of values), “trustworthiness and safety” (e.g., concerns about medical treatment abilities), and “others.” Of the total 982 codes, the majority focused on language barriers (723, 73.6%). Otherwise, a substantial number of participants indicated concerns about potential various issues due to gaps between Japan and foreign countries, such as differences in the sense of values (38, 3.9%), lack of understanding of Japanese characteristics (34, 3.5%), differences in customs (28, 2.9%), and cultural differences (24, 2.4%). Exemplar quotes are also shown in Table 4.

## Discussion

In this nationwide cross-sectional study, we examined Japanese laypeople’s nationality preferences for physicians and their expectations and concerns for care by foreign national physicians. We found that a considerable portion of the participants reported a preference for Japanese physicians with regard to various personal qualities, and to possible medical scenarios other than specialty expertise, general physical examination, and general ailments. In all personal qualities and possible medical scenarios other than taking a medical history, participants who had experience of being examined by a foreign national physician tended to express significantly fewer nationality preferences in physician qualities than those who did not have such experience. The number of concern codes (982) exceeded expectation codes (360), largely because many more participants provided concern comments (938) than expectation comments (337). Concerns were predominantly focused on language barriers, with a significant proportion also addressing potential challenges arising from social, cultural and related disparities between Japanese and foreign nationals. Participants also indicated expectations for the potential strengths of foreign physicians.

The quantitative analysis of our study revealed that Japanese laypeople show a significant preference for physicians of their own nationality over non-Japanese physicians. This result is consistent with substantial evidence from Western countries suggesting that patients prefer race-concordant physicians^18,19^. For instance, Gray and Stoddard revealed that 88% of whites reported receiving care from physicians of the same race/ethnicity, while 32% of minorities reported regular care from physicians of minority race/ethinicity^18^. Similarly, Saha et al. observed that approximately quarter of Blacks and Hispanic individuals with race-concordant physicians explicitly considered physician race/ethnicity when choosing their usual physician^19^. In our study, we found that approximately half to two-thirds of Japanese lay participants preferred nationality-concordant physicians, which is broadly comparable to the level reported in these U.S. studies. To our knowledge, this is the first study to identify a preference for social identity-concordant physicians in the Eastern context. Our findings are particularly noteworthy given Japan’s unique position of relatively high nationality homogeneity.

We speculate that these findings are attributable to several possible mechanisms: explicit/implicit bias and high-context culture. First, the observed pattern likely reflects explicit, consciously endorsed in-group preference by nationality^47^. The respondents directly indicated a nationality-concordant physician, consistent with public discourse in which “Japanese” and “foreign national” function as salient social categories^47^. Second, laypeople in Japan may also be susceptible to implicit bias, defined as unconscious attitudes or stereotypes that potentially lead to unfavorable evaluation of an individual based on their characteristics (e.g., gender, nationality, race)^48^. In Japan, where foreign national physicians account for a relatively small percentage of the total physician population, laypeople can have only limited exposure to healthcare providers from diverse nationalities. They may judge nationality-disconcordant physicians less favorably without being consciously aware of doing so. Third, Japan is characterized by its high-context culture, in which communication style heavily relies on social, cultural and historically-defined context^49^. Such a high-context culture invariably relies on the essential role of shared context and unspoken conversation cues^50^; in particular, a substantial portion of communication derives from non-verbal clues and shared social norms, which likely leads to a greater prevalence of intercultural communication issues compared to other contexts^51^. Consequently, laypeople may assume that foreign national physicians would be less capable of interpreting nuanced signs and communicating smoothly, even if their language skills are fluent.

Furthermore, this study yielded three notable findings. First, participants with prior experience of consulting a foreign national physician were significantly less likely to indicate a nationality-concordant preference (*p* < 0.01 for all physician quality items; *p* < 0.01 for all medical care scenarios except taking the medical history). This is consistent with intergroup contact theory, which proposes that increased contact between different groups can result in reduced bias and more inclusive attitudes^52^. In particular, such preferences are not fixed and are likely to alter through intergroup contact. Second, regarding specialty expertise, general physical examination, and general ailments, half of the lay participants reported no nationality preference. Nationality is less likely to affect their judgement when the clinical interaction is viewed as non-affective or non-invasive. Third, no significant disparities were observed in nationality preference regarding the taking of a medical history between those who had seen a foreign national physician and those who had not. This suggests that medical history taking, even without prior experience of being examined by a foreign-national physician, can be performed by foreign national physicians without raising nationality preference issues.

The integration of qualitative analysis with the quantitative analysis resulted in a more comprehensive and in-depth understanding of our findings. The content analysis revealed a marked asymmetry between expressed concerns and expectations. The dominant sub-theme among the expressed concerns was language barriers, followed by cultural incompatibility. These concerns are understandable given the unique characteristics of Japanese culture and the reputation of the Japanese language as one of the most challenging languages in the world to learn. This reputation is attributed to its complicated grammar, pronunciation, and Chinese script system (Kanji)^53,54^. However, graduates of foreign medical schools are required to pass a demanding screening process administered by the Japanese government and the Ministry of Health, Labour and Welfare, including the Japanese Language Medical Proficiency Test and National Medical Practitioners Qualifying Examination in Japan^55^. Consequently, this rigorous screening process ensures that successful applicants possess the necessary Japanese language proficiency and are eligible to practice medicine in Japan.

Some possible limitations of this study should be mentioned. First, the web survey study design carries a risk of sample bias. As described earlier, we employed quota sampling through the online survey panel. Chi-square tests showed several background differences between participants with and without prior experience of being examined by foreign-national physicians (e.g., region, educational attainment, frequency of medical visits, and usual use of university hospitals), which may have influenced their responses. Furthermore, online recruitment may have excluded laypeople without access to the Internet, thereby raising possible concerns for the representativeness of the sample and possibly limiting generalizability. Second, the questionnaire for our survey had not been validated. To address this concern, we developed the questionnaire with reference to previous studies^37–40^, and subsequently examined its reliability and validity against our dataset. Third, because our questionnaire did not specify the physician’s race/ethnicity, the study only assessed nationality-based preferences. Fourth, in our content analysis, the initial coding was conducted by a single researcher. Although all authors subsequently reviewed and refined the coding framework through iterative discussions, the absence of independent initial coding by multiple researchers may have introduced bias. Fifth, our sample did not include laypeople from foreign countries. Future studies are warranted to include them, and would provide in-depth insights on discourse regarding patient-physician interaction.

### Implications

Given Japan’s increasing globalization and the growing presence of IMGs^55^, our present findings have important implications for all relevant stakeholders. First, medical educators should proactively educate healthcare professionals—both Japanese and non-Japanese—to develop communication skills that address patient concerns that are likely and in large part shaped by explicit/implicit bias and cultural assumptions. This includes preparing foreign-national physicians to navigate Japan’s high-context communication style and assisting their cross-cultural adaptation^51^. Furthermore, the qualitative analysis in this study revealed the strengths that are expected of foreign national physicians. Clinical educators should support foreign national physicians such that these strengths can be utilized in the Japanese medical field. Second, public education is essential to addressing issues arising from stereotypes about physician nationality and the competence of foreign national physicians. Campaigns to increase the visibility of foreign national physicians and promote intergroup understanding can help reshape public attitudes towards foreign national physicians. Third, policy-makers should promote a more inclusive healthcare environment both for patients and healthcare professionals. This would include anti-discrimination protocols. A suitable multi-faceted approach will lead to a better healthcare system that values cultural responsiveness, diversity, equity, and inclusivity, and may ultimately result in better patient experience and outcomes.

## Conclusions

This study clarified the nationality preferences of laypeople regarding physician selection in Japan and the reasons behind these preferences. A substantial proportion of the laypeople reported a significant preference for Japanese over foreign national physicians. This finding is likely attributable to explicit/implicit bias and Japan’s high-context culture. Prior experience with consulting a foreign national physician was significantly associated with reduced nationality preference, indicating the potential for bias reduction through exposure. The qualitative analysis provided an in-depth understanding of lay participant perceptions, namely that their concerns predominantly centered on language barriers, as well as expectations about the potential strengths of foreign physicians. The study’s findings highlight the necessity of reconsidering medical education strategies, public education, and inclusive policy frameworks. These multifaceted approaches will address both patient and healthcare provider readiness for diversity, lead to an improved healthcare system that values diversity, equity, and inclusivity, and potentially result in enhanced patient experience and outcomes.

## Supplementary Information

Below is the link to the electronic supplementary material.

Supplementary Material 1

## Data Availability

The corresponding author can provide the data sets generated and analyzed in this study upon reasonable request.
