# Supplementary material for: Examining Japanese laypeople’s nationality preferences for physicians: a nationwide study
Source: Sci Rep. 2025 Nov 27;15:45527. doi: 10.1038/s41598-025-29740-6 (PMC12749866; doi:10.1038/s41598-025-29740-6)
Supplement: Supplementary file 1 — Supplementary Material 1 [file 41598_2025_29740_MOESM1_ESM.pdf]

Supplementary file: details of the survey

Q1. I freely give consent to take part in this study.

A1. Agree, Disagree

Q2. Are you a Japanese national?

A2. Yes, No, Others

Q3. Are you a healthcare staff?

A3. Yes, No

Q4. Your gender:

A4. Woman, Man, Others

Q5. Your age (year):

A5. 18–24, 25–34, 35–44, 45–54, 55–64, 65–74, ≥ 75

Q6. Your residential region:

A6. Hokkaido and Tohoku, Kanto, Chubu, Kinki, Chugoku and Shikoku, Kyushu

Q7. Your marital status:

A7. Never married, Married, Divorced or widowed

Q8. Your level of education:

A8. Less than high school, High school, Junior college, More than or equal to college

Q9. Your occupation:

A9. Manager or executive, Company employee, Self-employed, Public employee, Employee of other organizations, Part-time worker, Housewife/househusband, Student, Unemployed

Q10. Your annual household income (million JPY):

A10. < 3.00, 3.00–4.99, 5.00–6.99, 7.00–9.99, ≥ 10.00

Q11. Number of medical visits in the last six months:

A11. 0, 1, 2, 3, 4, 5–9, ≥ 10

Q12. Type of medical institution being usually attended:

A12. Clinic, Community hospital, University hospital

Q13. Number of foreign-national physicians you have been examined by so far:
A13. 0, 1, 2, 3, 4, 5–9, ≥ 10

The following questions (Q14–17) ask about preferences regarding physician qualities.
Q14. Empathy
A14. Prefer a Japanese physician, Prefer a non-Japanese physician, No preference

Q15. Specialty expertise
A15. Prefer a Japanese physician, Prefer a non-Japanese physician, No preference

Q16. Communication skill
A16. Prefer a Japanese physician, Prefer a non-Japanese physician, No preference

Q17. Good listener to the patient
A17. Prefer a Japanese physician, Prefer a non-Japanese physician, No preference

The following questions (Q18–24) ask about preferences for patient-physician interaction in possible medical scenarios.
Q18. Taking the medical history
A18. Prefer a Japanese physician, Prefer a non-Japanese physician, No preference

Q19. Discussing family or psychological problems (e.g., abuse, depression)
A19. Prefer a Japanese physician, Prefer a non-Japanese physician, No preference

Q20. General physical examination
A20. Prefer a Japanese physician, Prefer a non-Japanese physician, No preference

Q21. Examination with private body part exposure (e.g., clinical breast examination)
A21. Prefer a Japanese physician, Prefer a non-Japanese physician, No preference

Q22. General ailments (e.g., cold, stomach pain)
A22. Prefer a Japanese physician, Prefer a non-Japanese physician, No preference

Q23. Surgical operation
A23. Prefer a Japanese physician, Prefer a non-Japanese physician, No preference

Q24. Life-threatening conditions
A24. Prefer a Japanese physician, Prefer a non-Japanese physician, No preference

Q25. Please feel free to describe your expectations regarding patient care by foreign national physicians.

Q26. Please feel free to describe your concerns regarding patient care by foreign national physicians.
